# Food Delivery Apps and the Negative Health Impacts for Americans

**DOI:** 10.3389/fnut.2020.00014

**Published:** 2020-02-20

**Authors:** Janna Stephens, Hailey Miller, Lisa Militello

**Affiliations:** ^1^College of Nursing, Ohio State University, Columbus, OH, United States; ^2^School of Nursing, Johns Hopkins University, Baltimore, MD, United States

**Keywords:** smartphone, food delivery, applications, fast food, obesity

## Introduction

Food delivery applications have seen a surge in growth over the past decade. Digital ordering represents half of all food delivery visits, expanding beyond traditional dinner delivery to encompass breakfast and lunch delivery (1). Digital orders, ordered via a mobile app, Internet, or text message, have grown 23% over the past 4 years representing a $26.8 billion dollar industry (2). In most instances, digital food ordering can be done directly with a restaurant app or third party food service, which allow people to view local restaurants and menus (2). GrubHub, founded in 2004, was the first successful third-party food delivery system (3). The company rapidly grew, acquiring other online businesses and expanded into the delivery service arena. With approximately 44,000 restaurants on GrubHub's platform, food sales reported in October 2019 grossed about 1.4 billion dollars, an estimated 15% gross year-over-year increase (4). With their success came other services, like UberEats, which grew by 230% in 2017 (5). It is expected that food delivery applications will have over 44 million users in the United States in 2020 (6). While this growth has both positive and negative outcomes for restaurants, expanding their market yet costing them in fees, what does it mean for the American consumers and their health?

Roughly two-thirds of the American consumer utilizing a popular food-delivery platform, DoorDash, reported that food delivery was their preferred way of eating dinner (7). However, what many of these individuals might not realize is that the frequency of eating food from outside of the home is positively associated with a high body mass index (8). In a study done by Zion et al. (5), it was reported that 40% of people surveyed had used a multi-restaurant food delivery application in the past 90 days. Of those using the application services, 53% used it greater than 3 times in the past 3 months and of those, 7% had used it more than 11 times. Other data suggests that 10% of Americans use delivery services weekly and 52% typically order food delivery for lunch (9). When considering the increasing prevalence rates for overweight and obesity in the U.S., the effects of these digital food-delivery apps could be of great concern.

Overweight and obesity is a persisting epidemic in both pediatric and adult populations, with the most recent U.S. obesity (not including overweight) prevalence rates indicating that roughly 40% and 18% of adult and children are overweight or obese, respectively (10). In particular, the prevalence rate for obesity among young adults was reported to be 35.7% in 2016. Similarly, teens between 12-19 years of age had a reported obesity prevalence rate of 20.6% (10). These statistics are alarming considering that the majority users (63%) of food-delivery apps are youth adults ages 19–29 years of age (5).

It is well-established from longitudinal studies that that adolescents who have better diet quality gain less weight in adulthood compared to those with poorer diet quality (11), Due to diverse and competing food-delivery platforms, users have the potential to select healthy options when opting to use digital ordering. However, reports from the most frequently used platforms highlight that American consumers' top ordered foods include a cheeseburger and fries, pizzas, nachos, cheesecake, baby back pork rib, chicken and waffle sliders, etc., indicating that calorie-dense options are some of the most popular selections to be delivered (7). Other sources report that in 2016, pizza was the most popular takeout/delivery food, followed by Asian cuisine, sandwiches, and Italian cuisine (9). GrubHub noted that over 70% of users utilized their applications to order quick service or fast casual foods (12). Over the past 30 years, fast food portion sizes have increased, calories have increased, and sodium levels have increased, intensifying the potential problem these applications might pose to the ongoing obesity epidemic (8).

In addition to the frequent use in the young-adult age group, these food delivery services continue to rise in popularity across adolescent American students (13, 14). With up to 15 deliveries a day, school districts are reevaluating their policy and banning meal delivery services due to safety concerns (13, 14). A synopsis of school lunch delivery services that provide, “healthy,” “fresh,” “organic,” or “from scratch” foods average a cost of $4–$8 per entrée (15). This is a dramatic increase when compared to State School Nutrition data from 2017 highlighting lunch costs ranging from $2.48 to $2.74 per meal (16). In order to access more elite school lunch delivery services, schools must register with the service or parents must go online to coordinate/meal plan their child's food, or food service must align with National School Lunch Program standards (15). Meaning that access to healthy food deliveries is not equal across all student groups.

## Discussion

While we have a basic understanding of who is using these food delivery service applications and what they are ordering, there is currently no research to support how digital food ordering affects health and wellness on an individual level or from a public health perspective in the U.S. Are the most frequent users of these applications overweight or obese? If so, do these individuals have a desire to consume fewer calories and lose weight? If so, what behavior change techniques may be designed into digital ordering software to help promote health? The convenience of these applications may present a greater risk to adverse health outcomes among overweight or obese individuals, who consume more calories than their normal weight counterparts (17). Similarly, will rates of fast food consumption continue to increase among low-income individuals ages 20–39, who are known to consume a higher percentage of calories from fast food compared to those with higher income levels? (17).

These questions highlight critical gaps in the literature. We strongly advocate for unbiased additional research in this arena to objectively report on user demographics, wants, and needs in the United States. Considering the ease of digital food ordering, we also strongly advocate that digital food ordering platforms adapt ethical design to improve human situations, including health.

## Author Contributions

JS, LM, and HM assisted in the concept of the manuscript and the writing of all sections.

### Conflict of Interest

The authors declare that the research was conducted in the absence of any commercial or financial relationships that could be construed as a potential conflict of interest.
